# Metabolic changes and plasma glycine predict the risk of acute cellular rejection in heart transplantation

**DOI:** 10.1016/j.xjon.2026.101593

**Published:** 2026-01-21

**Authors:** Kishor Dhaygude, Richard S. Krebs, Rainer Krebs, SeoJeong Joo, Maria Hurskainen, Emil J. Holmström, Simo O. Syrjälä, Sangita Kulathinal, Antti I. Nykänen, Karl B. Lemström

**Affiliations:** a Translational Immunology Research Program and Transplantation Laboratory, Department of Pathology, Faculty of Medicine, University of Helsinki, Helsinki, Finland; b Division of Pediatric Cardiology, New Children's Hospital, Helsinki University Hospital and University of Helsinki, Helsinki, Finland; c Department of Cardiothoracic Surgery, Helsinki University Hospital and University of Helsinki, Helsinki, Finland; d Department of Mathematics and Statistics, University of Helsinki, Helsinki, Finland

**Keywords:** heart transplantation, brain death, metabolomics, plasma biomarkers, acute cellular rejection, glycine, donor organ quality

## Abstract

**Objective:**

Brain death in organ donors may trigger tissue injury that can negatively influence transplant outcome. We analyzed plasma metabolomic profiles in heart transplant donors and tested whether any donor metabolite may predict the risk of acute cellular rejection after heart transplantation.

**Methods:**

Plasma samples from 83 heart transplant donors and 20 healthy volunteers were profiled using quantitative targeted metabolomics of 102 metabolites. Plasma samples from another set of 48 heart transplant donors were used for validation.

**Results:**

The plasma levels of 24 metabolites representing 24% of the targeted metabolites were significantly altered in brain-dead heart transplant donors, compared with healthy controls. Alterations in the purine metabolism pathway were most prominent: adenine, xanthosine, allantoin, xanthine, and inosine monophosphate were upregulated, whereas adenosine monophosphate and adenosine were downregulated, indicating an energy metabolism shift and oxidative stress. Donor plasma glycine levels predicted the risk of acute cellular rejection (concordance = 0.74, area under the curve = 0.75; *P* < .01) in both the training and the validation cohort within 1-year after transplantation. Donor plasma glycine levels moderately correlated with other plasma metabolites linked to collagen formation and extracellular matrix organization.

**Conclusions:**

Targeted metabolomics revealed altered purine metabolism in brain-dead heart transplant donors, suggesting increased energy demand and oxidative stress. Donor plasma glycine was identified as a risk predictor for acute cellular rejection after heart transplantation and may serve as a donor-side biomarker to guide posttransplant risk stratification and monitoring.

## Graphical Abstract

**Figure:**
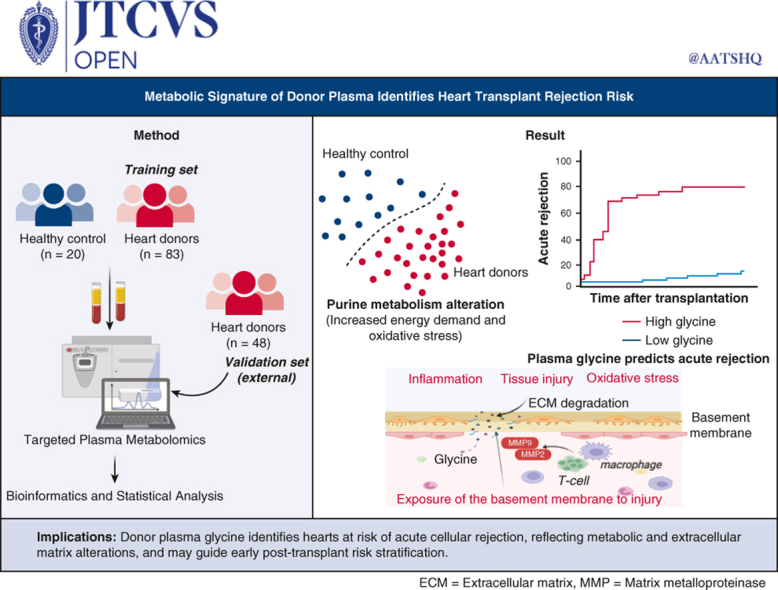

**Figure:**
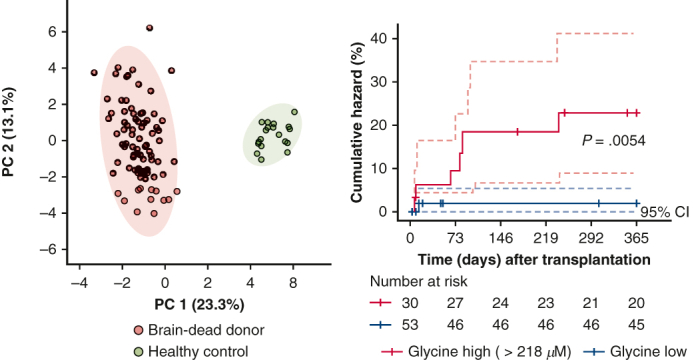
Plasma metabolomic alterations in heart transplant donors linked to acute rejection risk, with 95% CI.

**Central Message**

Plasma metabolite changes in heart transplant donors indicate shifts in energy metabolism, with glycine emerging as predictor of rejection risk and potential link to extracellular matrix remodeling.

**Perspective**

This study characterizes the plasma metabolomic profile of heart transplant donors to identify donor-derived signals linked to acute rejection. Targeted metabolomics revealed altered purine metabolism, and elevated glycine consistently predicted higher rejection risk. Donor plasma metabolites—especially glycine—may support early posttransplant risk stratification and more personalized monitoring.

Heart transplantation has been proven as a gold standard in the treatment of end-stage heart failure for selected patients. However, complications after heart transplantation, particularly acute cellular rejection, continue to influence graft survival and long-term outcomes. Identifying reliable biomarkers that predict rejection risk is critical for improving donor heart selection and optimizing the management of heart transplant recipients.

Brain death induces a cascade of physiological disturbances in organ donors, including metabolic and inflammatory shifts that can adversely affect the heart transplant immune response and allograft viability.^1^^,^^2^ However, the relationship between specific metabolic alterations in donor plasma and transplant rejection remains poorly understood. Metabolites, as intermediate and end products of cellular regulatory processes, offer insights into the response mechanisms of biological systems to genetic or environmental changes. The comprehensive and quantitative analysis of metabolites, metabolomics, has become a prevalent tool for the identification of diagnostic biomarkers.^3^, ^4^, ^5^, ^6^

This study aims to examine the plasma metabolomic profile of brain-dead heart transplant donors and explore the potential of donor plasma metabolites to identify hearts at risk of developing acute cellular rejection. We performed targeted metabolomic profiling of plasma from brain-dead heart transplant donors and compared it to healthy controls to identify metabolic markers linked to the risk of developing acute cellular rejection after heart transplantation. Our findings highlight alterations in purine metabolism in brain-dead donors compared with healthy controls, with donor plasma glycine emerging as a key metabolic signature associated with acute cellular rejection. These insights offer new possibilities for improving transplant outcomes through enhanced recipient risk prediction and better donor heart assessment.

## Methods

### Plasma Metabolomics of Heart Transplant Donors

We collected plasma samples from 84 brain-dead heart transplant donors in a prospective randomized clinical trial (Donor Simvastatin Treatment in Organ Transplantation [SIMVA]; ClinicalTrials.gov ID: NCT01160978) at Helsinki University Hospital (2010-2016).^7^ One donor sample was excluded due to inadequate plasma volume. The Finnish Red Cross Blood Service provided plasma samples from 20 age- and sex-matched healthy volunteers as controls. To validate our results, we used an external validation set of 48 heart transplantations (2019-2022) at the same hospital (Biomarkers for Diagnosis, Prognosis, and Targeted Therapy After Heart Transplantation [EMBIO]; ClinicalTrials.gov ID: NCT06064123). The study protocols were approved by the Institutional Review Board of Helsinki University Hospital for the SIMVA study (IRB No. 358/13/03/02/2009; ClinicalTrials.gov ID: NCT01160978; approved November 18, 2009) and the EMBIO study (IRB No. HUS/3654/2017; ClinicalTrials.gov ID: NCT06064123; approved December 13, 2017). All procedures were conducted in accordance with the Declaration of Helsinki. Consent was waived.

**Figure E1:**
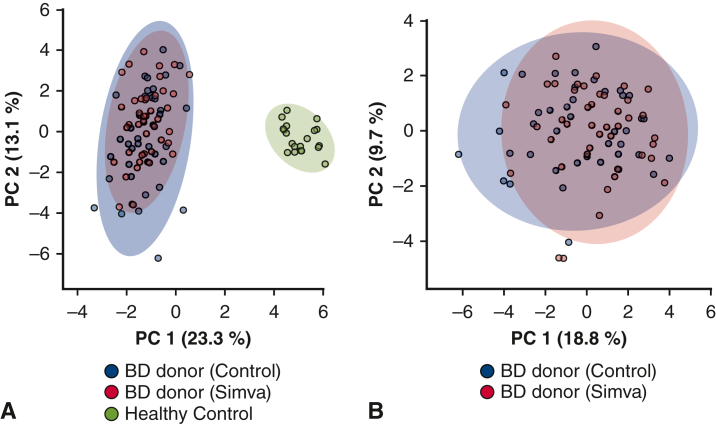
Simvastatin treatment did not significantly alter the plasma metabolic profile of heart donors. A, Principal component analysis (*PCA*) plot (principal component 1 [*PC1*] vs principal component 2 [*PC2*]) generated from the complete plasma metabolomics dataset, including simvastatin-treated brain-dead heart donors (Simva), untreated brain-dead heart donors (control), and healthy controls. The 2 brain-dead donor groups show overlapping metabolic distributions, whereas the healthy control group is clearly distinct. This indicates that simvastatin treatment does not produce major global metabolic shifts in donor plasma, whereas healthy controls exhibit a markedly different metabolic profile. B, PCA plot (PC1 vs PC2) restricted to heart donors only, comparing Simva and control. The 2 donor groups exhibit no clear separation along the PCs, further supporting that simvastatin exposure did not significantly influence the plasma metabolic profile in this cohort.

**Figure E2:**
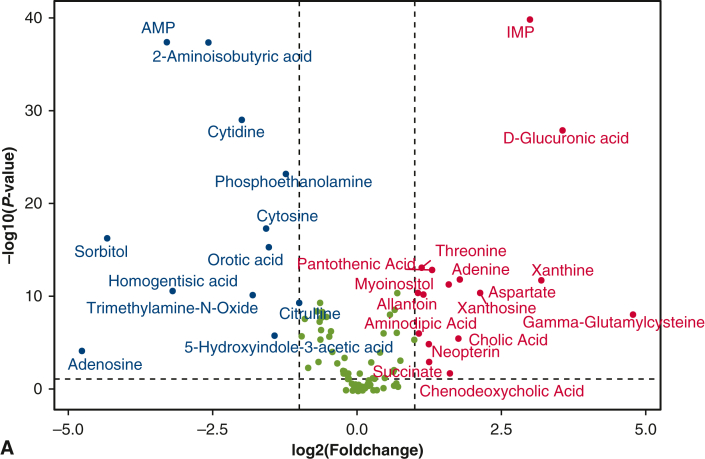

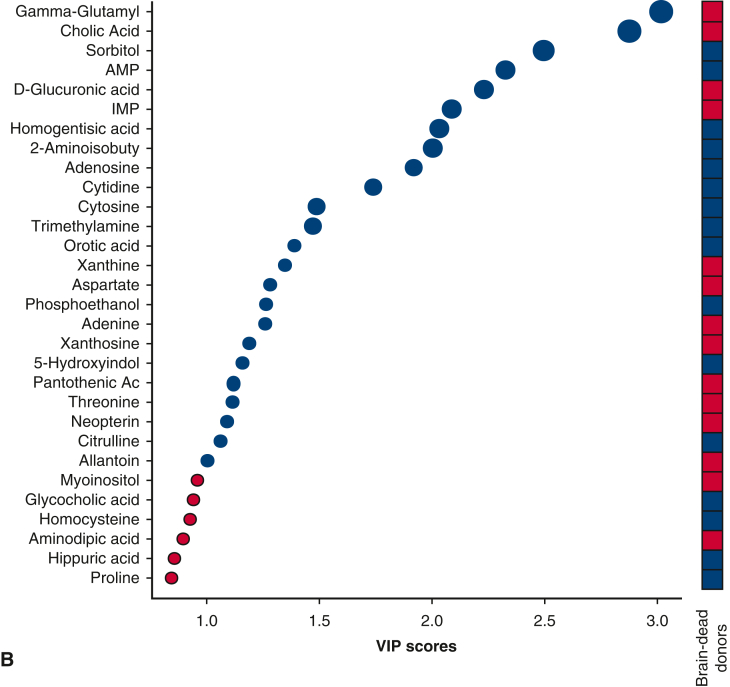
Univariate and multivariate analyses of significantly altered plasma metabolites in heart transplant donors. Targeted ultra-performance liquid chromatography-mass spectrometry was used to measure 102 plasma metabolites in brain-dead heart transplant donors (n = 83) and healthy controls (n = 20). Univariate and multivariate analysis were performed on 93 plasma metabolite levels found in both brain-dead heart transplant donors and healthy controls. A, A volcano plot displays significantly altered metabolites between brain-dead heart transplant donors and healthy controls, identified by univariate statistical analysis. It visually represents the distribution of metabolites showing significant differential expression between two groups. Metabolites with the fold change threshold of log2 >1 and the FDR corrected *P* value threshold < .05 were considered significant. Metabolites upregulated in brain-dead heart transplant donors compared with healthy controls are highlighted in red, whereas downregulated metabolites are shown in blue. *Greendots* are metabolites that did not differ significantly between the 2 groups. B, The supervised orthogonal partial least squares-discriminant analysis model was used to conduct multivariate analysis on 93 plasma metabolite levels found in both brain-dead heart transplant donors and healthy controls. Variable importance in projection values were used as a crucial metric for identifying significant metabolites. Metabolites with variable importance in projection values exceeding 1 were marked as significant, represented by dark blue filled circles in the analysis. The right panel displays metabolites upregulated in brain-dead heart transplant donors—compared with healthy controls—highlighted in red, whereas downregulated metabolites are shown in blue.

**Figure E3:**
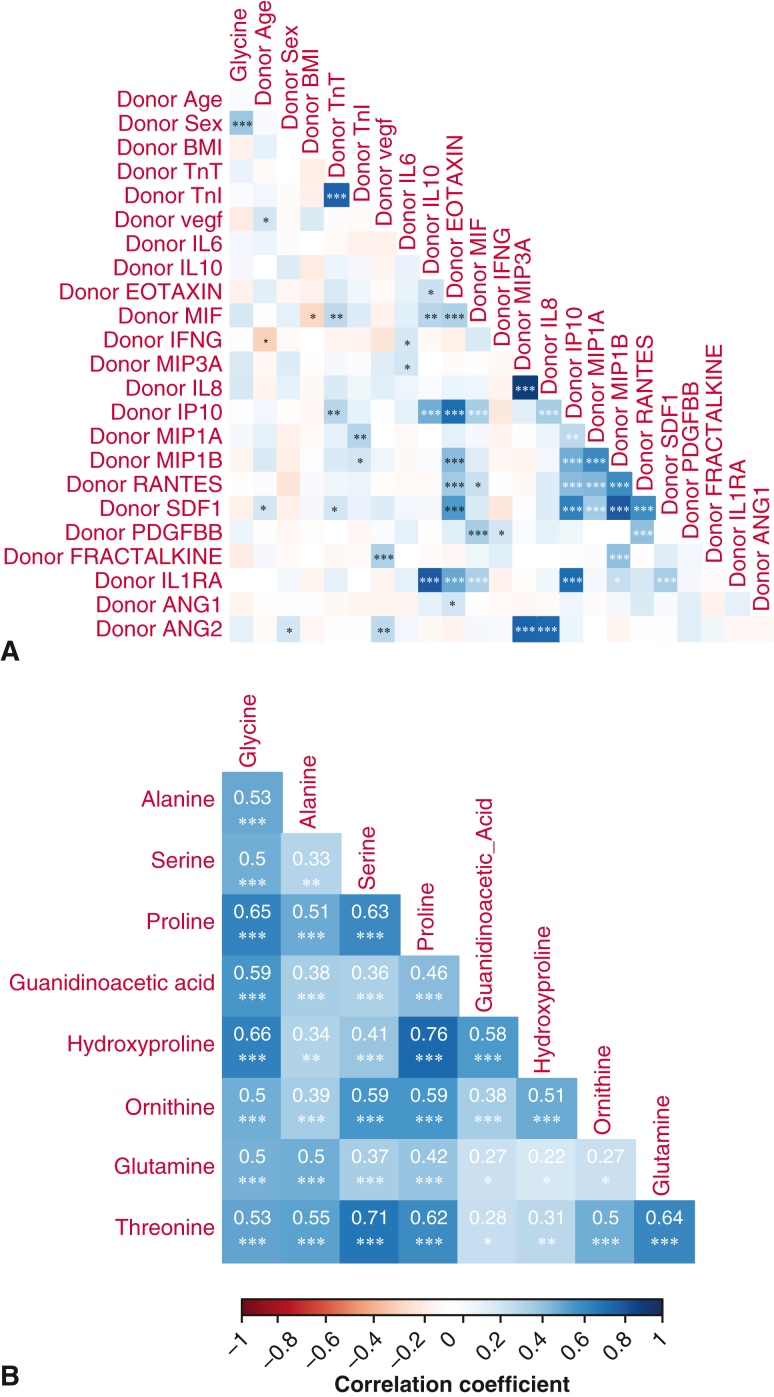
Correlation between heart transplant donor plasma glycine levels, demographics, cytokines, and metabolites. Donor plasma metabolites and cytokines were measured from 83 brain-dead donors using targeted ultra-performance liquid chromatography-mass spectrometry and multiplexed platforms, respectively. A, Correlation between heart transplant donor plasma glycine levels, donor demographic factors, and donor plasma cytokines. B, Correlation between heart transplant donor plasma glycine levels and other plasma metabolites. Correlation analysis was employed to calculate correlation coefficients (*r* value) and find the strength and direction of the association. The significance of the correlations was assessed using the *P* value. Positive correlations are displayed in blue and negative correlations in red. The intensity of the color and the values in the boxes reflect the Pearson correlation coefficients. ∗*P* < .05. ∗∗*P* < .01. ∗∗∗*P* < .001. *BMI*, Body mass index; *TnT*, troponin T; *TnI*, troponin I; *VEGF*, vascular endothelial growth factor; *IL-6*, interleukin-6; *IL-10*, interleukin-10; *EOTAXIN*, eotaxin (CCL11); *MIF*, macrophage migration inhibitory factor; *IFNG*, interferon gamma; *MIP-3A*, macrophage inflammatory protein-3 alpha (CCL20); *IL-8*, interleukin-8; *IP-10*, interferon gamma–induced protein 10 (CXCL10); *MIP-1A*, macrophage inflammatory protein-1 alpha (CCL3); *MIP-1B*, macrophage inflammatory protein-1 beta (CCL4); *RANTES*, regulated upon activation, normal T cell expressed and secreted (CCL5); *SDF-1*, stromal cell–derived factor 1 (CXCL12); *PDGF-BB*, platelet-derived growth factor BB; *FRACTALKINE*, fractalkine (CX3CL1); *IL-1RA*, interleukin-1 receptor antagonist; *ANG-1*, angiopoietin-1; *ANG-2*, angiopoietin-2.

**Figure E4:**
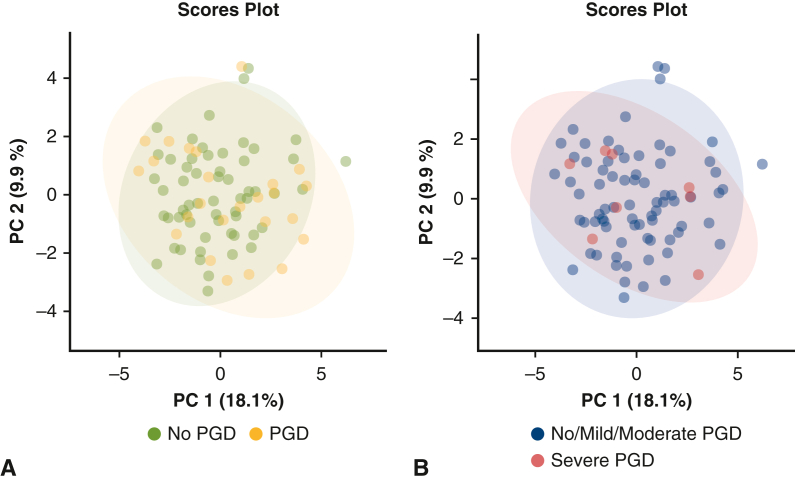
Lack of significant correlation between the plasma metabolite profile and the development of primary graft dysfunction. A, Principal component analysis plot (principal component 1 [*PC 1*] vs principal component 2 [*PC 2*]) generated from the full plasma metabolomics dataset, comparing donors who developed primary graft dysfunction (*PGD*) with those who did not (no PGD). The 2 groups show a substantial overlap, indicating that the global metabolite profile does not differentiate the PGD status. B, Comparison of donors with severe PGD versus donors with no, mild, or moderate PGD. The absence of clear separation between these categories further supports the lack of a strong association between plasma metabolite patterns and PGD severity.

Plasma samples were analyzed for metabolites using ultra-high-performance liquid chromatography coupled with mass spectrometry according to the manufacturer's instructions.^8^ In this technique, metabolites are first separated in a column based on their chemical properties and then identified and quantified by measuring their molecular mass. Data processing and statistical analysis were performed using MetaboAnalyst,^9^ including principal component analysis, partial least-squares discriminant analysis, orthogonal partial least-squares discriminant analysis, and standard univariate testing. Predictive models for acute rejection risk were built using Cox regression. Correlation analyses were conducted between glycine levels, other metabolites, and donor demographics. For details about the study design, donor management, plasma sample processing, definitions of clinical outcomes, and bioinformatics and statistical analyses, see Appendix E1, Figure E1, Figure E2, Figure E3, Figure E4.

## Results

### Heart Transplant Donor Demographics

**Figure 1:**
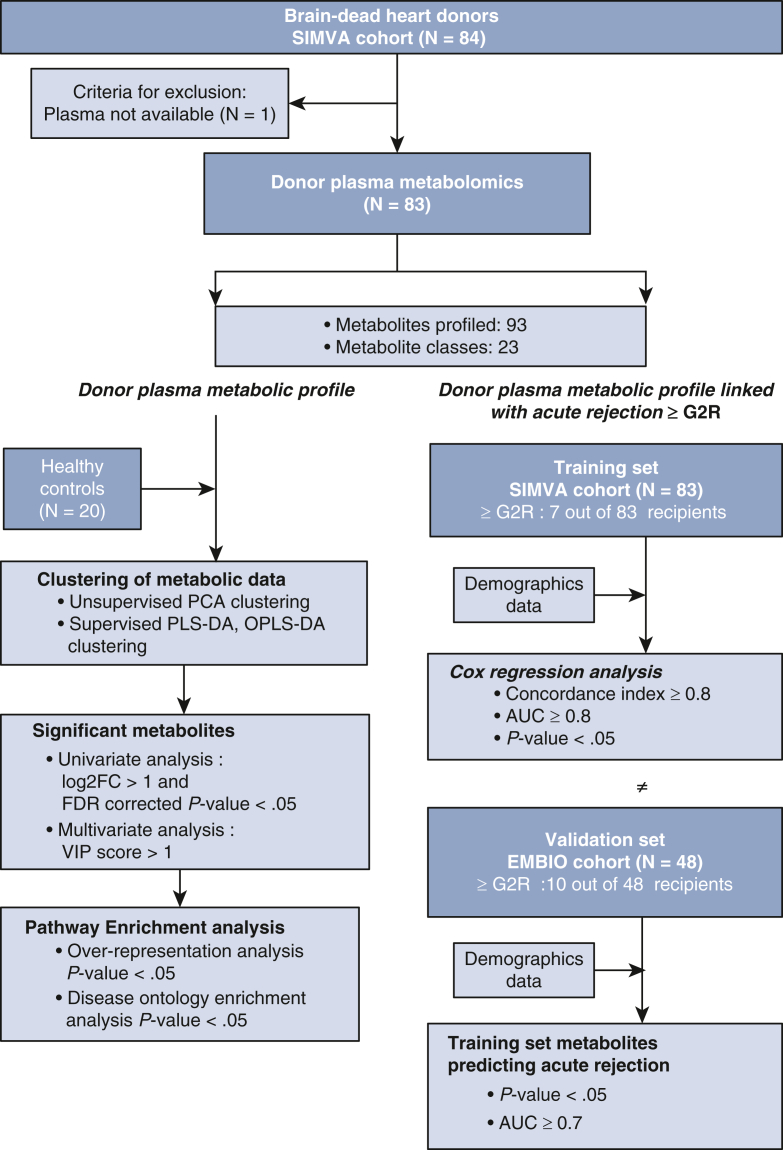
A consort-style flowchart showing patient sample flow through donor plasma metabolomics. Plasma samples were collected from 84 brain-dead heart donors participating in a prospective randomized clinical trial registered under Donor Simvastatin Treatment in Organ Transplantation [SIMVA] ClinicalTrials.gov (identifier NCT01160978). One donor sample was excluded due to inadequate plasma volume. Additionally, the study integrated plasma samples from 20 healthy volunteers, matched by age and sex to the study cohort, constituting the control group. The quantitative measurement of 93 out of the 102 targeted plasma metabolites was carried out using ultra-high performance liquid chromatography-mass spectrometry. We employed both unsupervised (principal component analysis [*PCA*]) and supervised (partial least squares discriminant analysis [*PLS-DA*] and orthogonal partial least squares discriminant analysis [*OPLS-DA*]) clustering methods to investigate whether the levels of the 93 metabolites displayed distinct separations between brain-dead heart transplant donors and healthy controls. Further analyses were conducted to find differentially expressed metabolites between brain-dead heart transplant donors and healthy controls, using univariate and multivariate statistical analyses. Significant metabolites found in these analyses were then used for pathway enrichment analysis. The subsequent phase focused on correlating the heart transplant donor plasma metabolomic profile with the occurrence of acute cellular rejection (Grade 2 rejection [≥G2R]). To predict and understand this association, brain-dead donor metabolomics data underwent a thorough analysis to find their association with the risk of developing biopsy-proven acute cellular rejection (≥G2R) within 1 year after heart transplantation. Cox regression analysis, a commonly employed statistical method for analyzing time-to-event data such as acute rejection with ≥G2R, was used. In our model, we included metabolite levels, donor age, and recipient age as predictive factors. The SIMVA cohort (n = 83) was chosen as the training set, whereas the Biomarkers for Diagnosis, Prognosis, and Targeted Therapy After Heart Transplantation (EMBIO) cohort (n = 48) served as the validation set. *AUC*, Area under the curve; *VIP*, variable importance in projection.

The final metabolomic analysis was performed on samples from 83 out of 84 brain-dead heart donors from the SIMVA cohort, and 20 healthy controls (Figure 1). The median age of heart donors was 44 years, and 24% were women. Eight (9.6%) heart donors had hypertension, and 13 (15.7%) heart donors received cardiopulmonary resuscitation at the time of brain injury (Table 1). The most common cause of brain death was cerebral bleeding, followed by traumatic brain injury, altogether accounting for 84.3% of deaths. The median age of the healthy controls was 45.5 years, and 35% were women.

**Table 1:** Baseline characteristics of the heart transplant donors and recipients

Characteristic	SIMVA cohort (n = 83)	EMBIO cohort (n = 48)	*P* value
Donor demographics			
Age (y)	44 (35.0-51.0)	42 (30.8-49.3)	.73
Female sex	20 (24.1)	8 (16.7)	.44
BMI	24.7 (23.0-26.3)	26.1 (23.8-29.2)	.02
Cause of death			
Intracranial hemorrhage	41 (49.4)	22 (46)	.9
Traumatic brain injury	29 (34.9)	16 (33)	
Cerebral infarction	5 (6.0)	3 (6.2)	
Cerebral anoxia after	4 (4.8)	4 (8.3)	
Other	4 (4.8)	3 (6.2)	
Smoking status			
Active	35 (42.2)	20 (41.6)	.24
Former	6 (7.2)	6 (12.5)	
Never	25 (30.1)	22 (45.8)	
Unknown	17 (20.5)	4 (8.3)	
Prior medical history			
Hypertension	8 (9.6)	7 (14.5)	.58
Diabetes mellitus	1 (1.2)	3 (6.2)	
EBV positive	76 (91.6)	39 (81.3)	.5
CMV positive	69 (83.1)	33 (68.8)	.06
Heart-related parameters			
Ejection fraction (%)	61 (60-65)	60 (55-65)	.31
Septum thickness (mm)	11.0 (10.0-12.0)	10 (9.7-11)	.49
Posterior wall thickness (mm)	10.0 (9.0-12.0)	9 (8.5-9.5)	.53
Inotropic support	74 (89.2)	29 (60.4)	
Predicted heart mass (g)	190.2 (178.9-213.3)	184 (162.2-196.0)	.13
Blood sample results			
Hemoglobin (g/L)	122 (107.5-133.7)	127 (113.7-138.5)	.33
Thrombocytes (E^9^/L)	172 (109-223)	213.5 (145.2-250.2)	.11
CRP (mg/L)	31 (9-93)	46 (8-112)	.31
CKMB (μg/L)	4.35 (2.5-13.8)	5 (3-12)	.88
TnI (ng/L)	42 (13.5-206)	24 (8-112)	.30
TnT (ng/L)	19.35 (9.3-48.0)	24.5 (13-121.3)	.36
Resuscitation	13 (15.7)	9 (16.7)	.81
Time of ROSC for resuscitated donors (min)	14.1 ± 11.3	19 ± 13.6	.73
Recipient demographics			
Age (y)	57 (46.5-61)	54.8 (44.4-61.5)	.52
Female sex	22 (26.5)	12 (25)	1
BMI	24.7 (22.5-28.0)	25.4 (23.2-27-2)	.6
Previous medical history			
Hypertension	11 (13.3)	8 (16.7)	.89
Diabetes mellitus	10 (12.0)	10 (20.8)	.41
Coronary artery disease	19 (22.9)	13 (27.1)	.74
COPD	3 (3.6)	0 (0.0)	1
Previous malignancy	7 (8.4)	0 (0.0)	1
Prior stroke	10 (12.0)	11 (22.9)	.22
Amiodarone <6 mo before transplantation	22 (26.5)	13 (27.1)	1
Prior sternotomy	24 (28.9)	26 (54.2)	.01
Smoking status			
Active	3 (3.6)	1 (2.1)	.65
Former	25 (30.1)	19 (39.6)	
Never	49 (59.0)	26 (54.2)	
Unknown	6 (7.2)	2 (4.2)	
CMV positive	62 (74.7)	36 (75)	1
Indication for HTx			
Nonischemic cardiomyopathy	42 (50.0)	17 (35.4)	.49
Coronary artery disease	19 (22.9)	13 (27.1)	
Myocarditis	6 (7.2)	1 (2.1)	
Sarcoidosis	5 (6.0)	6 (12.5)	
Congenital	3 (3.6)	3 (6.3)	
Hypertrophic cardiomyopathy	2 (2.4)	2 (4.2)	
Restrictive cardiomyopathy	1 (1.2)	1 (2.1)	
Other	5 (6.0)	3 (6.3)	
Retransplantation	0 (0.0)	1 (2.1)	
Organ functions before HTx			
PVR (WU)	2.5 (1.9-3.8)	2 (1.4-2.5)	.01
TPG (mm Hg)	9 (6.5-12.0)	10 (7.5-20.5)	.04
sPAP (mm Hg)	39.5 (30-50)	36 (24-43)	.17
P-bilirubin (μmol/L)	13 (10-22)	16.5 (12-31)	.07
Glomerular filtration rate (mL/min/1.73 m^2^)	54 (45-66.4)	56 (46-81)	.34
NT-proBNP (ng/L)	3300 (1234-6043)	2110 (1117-4860)	.13
MCS as a bridge to HTx			
LVAD, continuous flow	5 (6.0)	14 (29.2)	.01
LVAD, pulsatile flow	9 (10.8)	0 (0)	1
VA-ECMO	6 (7.2)	2 (4.2)	.48
High-urgency transplantation	6 (7.2)	5 (10.4)	.52
PRA I >20%	11 (13.3)	7 (14.6)	.83
PRA I >80%	2 (2.4)	0 (0.0)	1
PRA II >20%	4 (4.8)	6 (12.5)	.11
PRA II >80%	3 (3.6)	0 (0.0)	1
Recipient-donor sex mismatch			
Male-to-female	4 (4.8)	4 (8.3)	.67
Female-to-male	3 (3.6)	0 (0.0)	.73
Graft ischemia time (min)			
Total ischemia	180 (120-213)	184 (96-207)	.66
Recipient clinical outcomes			
Primary graft dysfunction			
Severe PGD	8 (9.6)	11 (22.9)	.04
Rejection			
1 mo rejection	18 (21.7)	11 (22.9)	1
1 y any rejection	51 (61.4)	22 (45.8)	.08
1 y >G2R rejection	7 (8.4)	10 (20.8)	.08
Survival			
1 mo	78 (94.0)	46 (95.8)	.96
1 y	71 (85.5)	42 (87.5)	.96

Values are presented as median (interquartile range) or n (%). *SIMVA*, Donor Simvastatin Treatment in Organ Transplantation; *EMBIO*, Biomarkers for Diagnosis, Prognosis, and Targeted Therapy After Heart Transplantation; *BMI*, body mass index; *EBV*, Epstein-Barr virus; *CMV*, cytomegalovirus; *CRP*, C-reactive protein; *CKMB*, creatine kinase-MB; *TnI*, high-sensitivity troponin I; *TnT*, high-sensitivity troponin T; *ROSC*, return of spontaneous circulation; *COPD*, chronic obstructive pulmonary disease; *HTx*, heart transplantation; *PVR*, pulmonary vascular resistance; *TPG*, transpulmonary gradient; *sPAP*, systolic pulmonary arterial pressure; *NT-proBNP*, N-terminal prohormone of brain natriuretic peptide; *MCS*, mechanical circulatory support; *LVAD*, left ventricular assist device; *VA-ECMO*, veno-arterial extracorporeal membrane oxygenation; *PRA*, panel reactive antibody; *PGD*, primary graft dysfunction; *G2R*, Biopsy-proven grade 2 rejection.

### Heart Transplant Donors Showed a Distinct Plasma Metabolomic Profile

**Figure 2:**
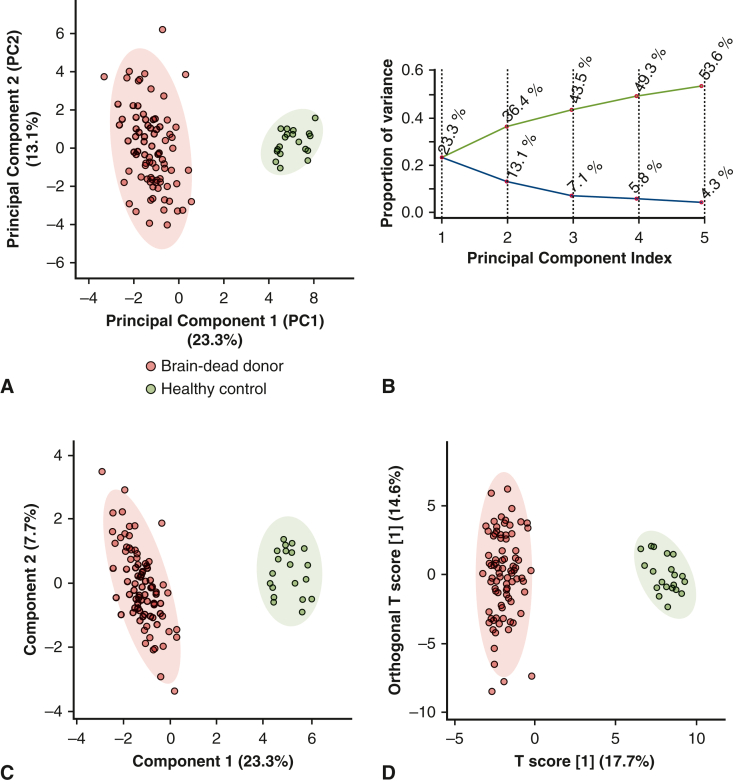
Unsupervised and supervised clustering analyses of plasma metabolomic profiles in brain-dead heart transplant donors and healthy controls. Targeted ultra-high performance liquid chromatography–mass spectrometry (*UHPLC-MS*) was used to measure 93 out of the 102 targeted plasma metabolites in brain-dead heart transplant donors (n = 83) and healthy controls (n = 20). A, Unsupervised principal components analysis clustering between brain-dead heart transplant donors (*red dot*) and healthy controls (*green dot*). B, The scree plot showed the proportion of variance explained by each principal component. Supervised clustering analysis using predefined group labels with partial least squares discriminant analysis (C) and orthogonal partial least squares discriminant analysis (*OPLS-DA*) (D) models. The metabolomics data was normalized using the median approach, log2 transformation, and pareto scaling. Score plots (A, C, and D) showed normalized data of metabolite levels within the 95% confidence ellipses and detected no clear outlier in either group. In these plots, principal component 1 (*PC1*) and principal component 2 (*PC2*) represent composite axes that capture the largest and second-largest sources of variation in the metabolite data, respectively. In the OPLS-DA model, the T score indicates each sample's position along a latent variable that best separates donors from controls.

Plasma samples from brain-dead heart donors (n = 83) of the SIMVA cohort and from healthy controls (n = 20) yielded signals for 93 out of 102 (91.2%) targeted metabolites by ultra-high-performance liquid chromatography coupled with mass spectrometry. These metabolites consisted of amino acids, bile acids, acylcarnitines, enzyme cofactors, choline metabolites, nucleotides, and nucleic acids (Table E1). Unsupervised PCA demonstrated distinct clustering of brain-dead donors and healthy controls (Figure 2, *A* and *B*). Supervised clustering analyses, including partial least-squares discriminant analysis (Figure 2, *C*) and orthogonal partial least-squares discriminant analysis (Figure 2, *D*), further confirmed these findings.

To find differentially expressed metabolites, all 93 metabolites were subjected to univariate and multivariate statistical analyses. With the cutoff criteria for fold-change of >1 and an false discovery rate-corrected *P* value < .05, the univariate analysis revealed that 28 metabolites (30.1%) had significantly different plasma levels between heart transplant donors and healthy controls (Table 2 and Figure E2). When subjected to multivariate analysis with a variable importance in projection score >1, 24 out of the 28 metabolites identified in univariate analyses (25.8%) had also significantly different plasma levels between heart transplant donors and healthy controls (Table 2). The full list of differentially expressed metabolites is shown in Table 2.

**Table 2:** Differentially altered metabolites between heart transplant donors and healthy controls found using univariate (volcano plot) and multivariate (PLS-DA) analysis∗

Metabolite	HMDB ID	log2(FC)	*P* value	VIP score	Classes	Significant method
Upregulated						
Gamma-glutamylcysteine	HMDB0001049	4.78	6.91^E-30^	3.01	Amino acid	†
D-glucuronic acid	HMDB0000127	3.56	2.76^E-28^	2.22	Other	†
Xanthine	HMDB0000292	3.19	8.74^E-06^	1.34	Purine and pyrimidine	†
IMP	HMDB0000175	2.99	3.34^E-31^	2.08	Purine and pyrimidine	†
Xanthosine	HMDB0000299	2.13	5.91^E-07^	1.19	Purine and pyrimidine	†
Adenine	HMDB0000034	1.78	2.37^E-10^	1.26	Purine and pyrimidine	†
Cholic acid	HMDB0000619	1.76	2.12^E-23^	2.87	Bile acids	†
Chenodeoxycholic acid	HMDB0000518	1.61	2.08^E-02^	0.64	Bile acids	‡
Aspartate	HMDB0000191	1.59	1.30^E-12^	1.28	Amino acid	†
Pantothenic acid	HMDB0000210	1.30	4.00^E-11^	1.12	Fatty acids, conjugates	†
Succinate	HMDB0000254	1.25	4.41^E-02^	0.44	Tricarboxylic acid cycle	‡
Neopterin	HMDB0000845	1.24	4.08^E-09^	1.09	Other	†
Allantoin	HMDB0000462	1.15	2.96^E-09^	1.01	Purine and pyrimidine	†
Threonine	HMDB0000167	1.12	4.54^E-14^	1.11	Amino acid	†
Aminodipic acid	HMDB0000510	1.07	7.72^E-07^	0.89	Fatty acids, conjugates	‡
Myoinositol	HMDB0000211	1.05	4.06^E-09^	0.96	Benzamides	‡
Downregulated						
Adenosine	HMDB0000050	−4.76	4.23^E-13^	1.91	Purine and pyrimidine	†
Sorbitol	HMDB0000247	−4.33	3.00^E-17^	2.49	Monosaccharides	†
AMP	HMDB0000045	−3.29	2.90^E-22^	2.32	Purine and pyrimidine	†
Homogentisic acid	HMDB0000130	−3.19	7.92^E-14^	2.03	Other	†
2-Aminoisobutyric acid	HMDB0001906	−2.57	4.24^E-28^	1.99	Amino acid	†
Cytidine	HMDB0000089	−2.00	1.06^E-22^	1.73	Glycosyl compounds	†
Trimethylamine-N-oxide	HMDB0000925	−1.81	1.88^E-09^	1.47	Amine oxide	†
Cytosine	HMDB0000630	−1.57	9.05^E-16^	1.48	Purine and pyrimidine	†
Orotic acid	HMDB0000226	−1.53	1.21^E-11^	1.38	Purine and pyrimidine	†
5-Hydroxyindole-3-acetic acid	HMDB0000763	−1.43	2.07^E-08^	1.16	Indoles	†
Phosphoethanolamine	HMDB0000224	−1.23	2^E-13^	1.26	Other	†
Citrulline	HMDB0000904	−1.00	2.65^E-09^	1.06	Amino acid	†

*HMDBID*, Human Metabolome Database identifier; *log2(FC)*, log_2_ fold change; *VIP*, variable importance in projection; *IMP*, inosine monophosphate; *AMP*, adenosine monophosphate.

∗ Univariate analysis with a fold-change threshold of >1 and an FDR corrected *P* value < .05 is considered significant, whereas multivariate analysis with a VIP score >1 is considered significant.

† Metabolites significant in both univariate and multivariate analyses.

‡ Metabolites significant in only the univariate analysis.

### Significantly Altered Metabolites in Heart Transplant Donors Were Related to Purine Metabolism

The pathway analysis results showed that these differently expressed metabolites were enriched in 4 pathways, namely purine metabolism, ascorbate and aldarate metabolism, arginine biosynthesis, and pantothenate and coenzyme A biosynthesis (Table 3).

**Table 3:** Kyoto Encyclopedia of Genes and Genomes (KEGG) pathway and Disease Ontology Semantic and Enrichment (DOSE) database disease-associated enrichment of significantly changed metabolite levels in heart transplant donors

Analysis	Enriched terms	*P* value	Upregulated metabolites in donors	Downregulated metabolites in donors	Selection bias∗
Pathways- associated terms	Purine metabolism	3.44^E-05^	Adenine, xanthosine, allantoin, xanthine, IMP	Adenosine monophosphate, Adenosine	No
	Ascorbate and aldarate metabolism	6.19^E-03^	myo-inositol, D-glucuronic acid		Yes
	Arginine biosynthesis	1.90^E-02^	Aspartate	Citrulline	Yes
	Pantothenate and CoA biosynthesis	3.40^E-02^	Pantothenic acid, aspartate		Yes
Disease- associated terms	Early markers of myocardial injury	6.52^E-04^	Xanthine, L-threonine	2-Aminoisobutyric acid, trimethylamine N-oxide	NA
	Extrahepatic biliary atresia	3.03^E-03^	Chenodeoxycholic acid, cholic acid		NA
	Refractory localization-related epilepsy	3.10^E-03^	Aspartate, L-threonine	Citrulline	NA
	Different seizure disorders	5.66^E-03^	Aspartate, , L-threonine	Citrulline, 5-hydroxyindoleacetic acid	NA

*IMP*, Inosine monophosphate; *CoA*, coenzyme A; *NA*, not available.

∗ To rule out the possibility of selection bias in targeted metabolites, we performed an overrepresentation analysis on 93 identified metabolites as a reference set.

Pathway enrichment analysis identified purine metabolism as a significantly altered pathway in donor plasma. To assess whether our targeted metabolite panel biased the representation of specific pathways, we performed an overrepresentation analysis using all 93 detected metabolites as the reference set. This showed that purine metabolism was the only pathway for which the detected metabolites reflected the pathway as a whole, rather than being strongly influenced by the predefined list of targeted metabolites. In the purine metabolism pathway, metabolites such as adenine, xanthosine, allantoin, xanthine, and inosine monophosphate were upregulated in brain-dead donors, whereas adenosine monophosphate and adenosine were downregulated (Table 3).

Furthermore, we used the Disease Ontology Semantic and Enrichment database to annotate the associations of the significant metabolites with diseases. We found that the metabolite sets were enriched in 4 disease-associated categories, namely early markers of myocardial injury, extrahepatic biliary atresia, refractory localization-related epilepsy, and different seizure disorders (Table 3).

### Donor Plasma Glycine, Glyceraldehyde, Phenylalanine, and Threonine Levels Predicted the Risk of Acute Cellular Rejection of Heart Transplant

Next, we studied whether plasma metabolites associated with the pathophysiological shifts in brain-dead heart transplant donors could potentially serve as predictive markers for identifying those donor hearts that are at risk of experiencing biopsy-proven acute cellular rejection of grade ≥G2R within 1 year after transplantation. The median age of heart transplant recipients was 57 years, with 26% of recipients being women. The main indication for heart transplantation was nonischemic dilated cardiomyopathy (50%). Among the 83 patients in the training set, 7 recipients (8.4%) experienced biopsy-proven acute cellular rejection (≥G2R) within 1 year after transplantation. Notably, all these rejections (≥G2R) occurred within the first 3 months (<97 days) posttransplant, except for 1 patient who experienced acute cellular rejection at 237 days.

**Figure 3:**
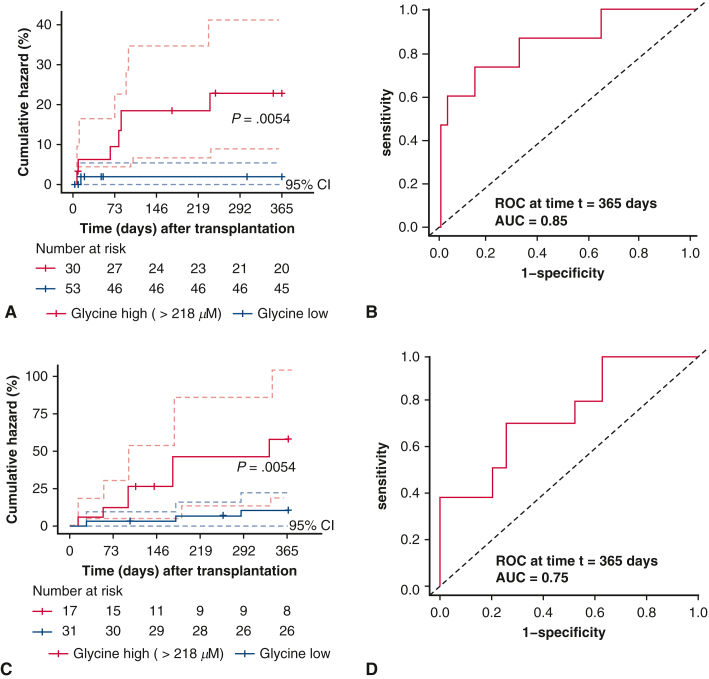
Performance of heart transplant donor plasma glycine levels in predicting the risk of acute cellular rejection. Targeted ultra-high performance liquid chromatography-mass spectrometry was used to measure plasma glycine levels in brain-dead heart transplant donors (n = 83). Donors were stratified into high and low glycine groups using the maximally selected rank statistics (*maxstat*) algorithm. High glycine was defined as concentrations above the data-driven optimal threshold of 218 μM. Optimal cut points for each continuous variable were determined to maximize outcome separation (log-rank statistic), whereas correcting for multiple testing. The maximally selected rank statistics algorithm was used to stratify heart transplant donors into 2 groups based on high and low plasma glycine levels. Cumulative hazard of risk of acute rejection ≥G2R (A) and receiver operating characteristics (*ROC*) curve (B) for predicted model for the training set (n = 83) cohort. Cumulative hazard of risk of acute rejection ≥G2R (C) and ROC curve (D) for predicted model for the validation set (n = 48) cohort. The area under the curve (*AUC*) value for ROC curve was calculated at 1 year after heart transplantation. Cumulative hazard curves for ≥G2R acute cellular rejection are shown, with the *y*-axis expressed as percentages (0%-100%) and the *x*-axis in days to reflect the 1-year follow-up period. The numbers of subjects at risk are displayed at appropriate intervals along the timeline, and 95% CI are indicated by dotted lines.

Based on the donor plasma levels of 93 metabolites, Cox regression analysis revealed that 12 metabolites were associated with biopsy-proven acute cellular rejection of grade ≥G2R within 1 year (Table E2). However, when we tested the quality of the prediction model with more stringent criteria including concordance ≥0.8, area under the curve ≥0.8, and false discovery rate-corrected *P* value < .05 cutoff values, only glycine, glyceraldehyde, phenylalanine, and threonine were found to be associated with biopsy-proven acute cellular rejection of grade ≥G2R within 1 year (Table 4). Using maximally selected rank statistics (*maxstat*), we determined the optimal data-driven cutoff value for each of these four metabolites and stratified donors into high and low plasma level groups. High glycine was defined as concentrations above the cohort median (218 μM), and the optimal *maxstat*-derived threshold closely aligned with this median-based categorization (Figure 3, *A* and *B*). This analysis revealed that higher donor plasma levels of all 4 metabolites were associated with an increased risk of developing biopsy-proven acute cellular rejection with grade ≥G2R within 1 year.

**Table 4:** Influence of plasma metabolite levels in heart transplant donors on the risk of developing acute rejection ≥G2R after heart transplantation

Metabolite	Event	Concordance	Hazard ratio (95% CI, *P* value)	Area under the curve∗	Area under the curve†
Training set (n = 83)					
Glycine	7	0.81 ± 0.082	1.02 (1.01-1.02; *P* < .001)	0.85	0.81
Glyceraldehyde	7	0.88 ± 0.047	1.04 (1.02-1.06; *P* < .001)	0.88	0.86
Phenylalanine	7	0.84 ± 0.057	1.06 (1.03-1.10; *P* = .001)	0.85	0.80
Threonine	7	0.81 ± 0.067	1.02 (1.01-1.03; *P* < .001)	0.84	0.80
Validation set (n = 48)					
Glycine	10	0.74 ± 0.07	1.01 (1-1.02; *P* = .012)	0.75	0.73
Glyceraldehyde	10	0.67 ± 0.09	1.01 (1-1.02; *P* = .120)	0.58	0.59
Phenylalanine	10	0.67 ± 0.1	1.04 (0.99-1.09; *P* = .167)	0.59	0.67
Threonine	10	0.62 ± 0.09	1.01 (0.99-1.02; *P* = .299)	0.60	0.64

Values are presented as concordance ± SE unless otherwise noted.

*G2R*, Grade 2 rejection.

∗ Time-dependent receiver operating characteristic, *t* = 365.

† Weibull model, *t* = 365.

### Validation Set Confirmed Only Donor Plasma Glycine as a Predictor for Acute Cellular Rejection of Heart Transplant

To validate the 4 candidate metabolites associated with biopsy-proven acute cellular rejection of grade ≥G2R within 1 year after heart transplantation, we used an external validation set including 48 consecutive heart transplantations in the EMBIO cohort. The median age of the heart transplant donors was 42 years, and 17% were women (Table 1). The median age of the heart transplant recipients was 55 years, and 25% were women. In this cohort, the main indication for heart transplantation was also nonischemic dilated cardiomyopathy (35%). Out of these 48 patients, 10 recipients (20.8%) experienced biopsy-proven acute cellular rejection ≥G2R within 1 year. Based on continuous heart transplant donor plasma metabolite levels, we found that only high glycine levels were significantly associated with biopsy-proven acute cellular rejection of grade ≥G2R within 1 year (Table 4 and Figure 3, *C*). The significance of glycine was confirmed with a concordance of 0.74, area under the curve of 0.75, and *P* < .01 (Figure 3, *D*). Donor plasma glycine levels were associated specifically with the risk of acute cellular rejection (≥G2R) but showed no correlation with either short- or long-term transplant survival or with primary graft dysfunction, indicating that these end points were not influenced by glycine levels in either the training or validation cohorts.

### Glycine Correlated With Extracellular Matrix Organization-related Metabolites Proline and Hydroxyproline

Earlier studies have suggested a correlation between brain injury and high systemic glycine levels.^10^, ^11^, ^12^ The brain injury may trigger systemic inflammatory response and thereby enhance the ischemic injury and immune response in donor hearts. Consequently, this cascade could increase the risk of acute cellular rejection.^1^^,^^13^^,^^14^ First, we investigated the correlations between heart transplant donor plasma glycine levels, demographics, and cytokines levels within the SIMVA cohort. Our findings revealed a weak correlation between donor sex and glycine levels, with female donors having higher plasma glycine levels compared to male donors (Figure E3). However, donor sex did not show any association with the risk of acute cellular rejection (≥G2R) within the first year after heart transplantation.

Moreover, glycine is the most abundant amino acid within collagen, which is a crucial component of the cardiac extracellular matrix. Studies suggest that brain death may trigger the degradation and remodeling of this matrix.^15^, ^16^, ^17^ Although we did not find any correlation between heart transplant donor plasma glycine levels and donor heart myocardial injury, as measured by donor plasma high sensitivity troponin levels (Figure E3, *A*), we did see a moderate correlation between heart donor plasma glycine levels and collagen-associated metabolites such as proline, hydroxyproline, and serine. Additionally, donor plasma glycine levels were related to other metabolites such as alanine, guanidinoacetic acid, ornithine, glutamine, and threonine (Figure E3, *B*).

## Discussion

This study demonstrated significant metabolomic changes in brain-dead heart transplant donors compared with healthy controls. We observed changes in 24 donor plasma metabolite levels spanning across 4 pathways, including purine metabolism, ascorbate and aldarate metabolism, arginine biosynthesis, and pantothenate and coenzyme A biosynthesis. Furthermore, the results suggested that elevated plasma glycine levels in brain-dead donors may serve as a biomarker for predicting the risk of acute rejection within the first year after transplantation. These findings indicate that donor plasma metabolites could be crucial for assessing donor heart quality, paving the way for improved care strategies for heart transplant recipients.

We found that most of the altered donor plasma metabolites belonged to purines-pyrimidines and amino acids biochemical classes. These compounds play central roles in the regulation of energy production and utilization in the cell.^18^ Of the purine-pyrimidine pathway-related metabolites, inosine monophosphate, xanthine, and allantoin were increased, whereas adenosine, adenosine monophosphate, cytosine, and orotic acid were reduced in brain-dead heart donors. This shift in the purine-pyrimidine pathway suggests enhanced purine catabolism to maintain energy supply and oxidative stress.^19^, ^20^, ^21^ The increased energy demand and oxidative stress may reflect the inflammation and tissue injury in brain-dead donors.^22^^,^^23^

This study also revealed elevated plasma levels of amino acids such as gamma-glutamyl cysteine, aspartate, and threonine, whereas the plasma levels of 2-aminoisobutyric acid, and citrulline were reduced. These amino acids are involved in various essential cell functions.^24^, ^25^, ^26^, ^27^ Of particular significance, gamma-glutamyl cysteine serves as a precursor in the biosynthesis of an essential antioxidant, glutathione.^28^ In addition, changes in the plasma levels of citrulline and aspartate may indicate disrupted arginine biosynthesis, which usually supports wound healing, nitric oxide production, protein synthesis and immune function.^29^^,^^30^

In the prediction analysis of the training set, donor plasma levels of glycine, glyceraldehyde, phenylalanine, and threonine were significantly associated with the development of biopsy-proven acute cellular rejection within the first year. When these results were tested in another cohort of heart transplant recipients, only glycine remained significant. The difference in these results may be partly explained by the different anticoagulants used in the training and validation sets. Glycine is a fundamental nonessential amino acid and participates in the synthesis of critical molecules such as purines, heme, creatine, glutathione, and collagen.^31^, ^32^, ^33^ Glycine comprises approximately one-third of collagen, a major structural component of the extracellular matrix.^15^, ^16^, ^17^^,^^34^ Elevated glycine levels in donor plasma may therefore reflect increased collagen breakdown or myocardial tissue injury in brain-dead donors. Elevated glycine could also reflect donor cellular injury—such as mitochondrial dysfunction, redox imbalance, or 1-carbon metabolic perturbations—or a proteolytic/inflammatory milieu, both of which may enhance innate immunity and prime alloimmune responses.^35^, ^36^, ^37^ Consistent with this hypothesis, donor glycine levels showed no association with rejection risk in kidney transplant recipients (Salin and colleagues, unpublished data), suggesting a heart-specific source of elevated glycine. These findings highlight donor glycine as a potential biomarker of cardiac injury in the context of heart transplantation, but we do not propose its use for donor-recipient matching or organ allocation at this stage. Instead, donor glycine should be viewed as a promising donor-side risk-stratification biomarker that may help to identify recipients who could benefit from closer early surveillance and careful avoidance of premature immunosuppression minimization. Larger multicenter studies are required to validate its predictive performance and to evaluate cost-benefit before clinical use.

## Conclusions

Our study found significant metabolic alterations in the plasma of brain-dead donors, primarily related to energy metabolism and amino acid biosynthesis. Additionally, we identified plasma glycine levels in heart transplant donors as a potential biomarker for predicting the risk of developing acute cellular rejection after heart transplantation. This association was specific to heart transplant recipients because no such relationship was observed in kidney transplant recipients. The correlation between glycine and other metabolites involved in extracellular matrix organization suggests a connection to myocardial tissue remodeling or injury. These results highlight the critical importance of identifying metabolic biomarkers, which could help clinicians assess donor-specific risk for acute cellular rejection. Further investigation of metabolic biomarkers in larger patient clinical trials is necessary to validate their clinical utility.

### Limitations

Our study is subject to several limitations that warrant careful consideration. Firstly, our use of a targeted metabolomics approach, while providing valuable insights, may have restricted our ability to comprehensively capture the entirety of metabolic processes. Therefore, a broader metabolomics approach could potentially offer a more extensive understanding. Secondly because our metabolomics study primarily focused on plasma samples, it does not provide a detailed molecular insight into organ- or tissue-specific metabolic alterations. Consequently, our ability to precisely identify the origins of glycine is constrained. Thirdly, an explanation for the disparity between the training and validation sets in prediction analyses could stem from the potential influence of different anticoagulants used for plasma collection. Although direct evidence linking anticoagulants to observed metabolic alterations is lacking, they can inadvertently affect metabolomics studies. These agents might augment metabolite ionization, induce matrix effects, or introduce chemical noise, all potentially interfering with correct metabolite quantification. Lastly, the critical illness and medication exposure of brain-dead donors might have influenced the plasma metabolomic profiles, complicating the interpretation of the results. However, these donors were excellent heart donors, potentially minimizing the influence of diverse treatment strategies. In addition, the validation cohort was of moderate size, and both cohorts were from a single center, which may limit generalizability and underscores the need for multicenter validation.

## Conflict of Interest Statement

The authors reported no conflicts of interest.

The *Journal* policy requires editors and reviewers to disclose conflicts of interest and to decline handling or reviewing manuscripts for which they may have a conflict of interest. The editors and reviewers of this article have no conflicts of interest.

## Figures and Tables

**Table E1:** The selected targeted metabolites come from 23 different classes, covering a wide range of metabolic pathways

Class and metabolite name	HMDB ID	Measured in study
Alpha amino acids and derivatives		
2-Aminoisobutyrate	HMDB0001906	Yes
4-l-Hydroxyproline	HMDB0000725	Yes
5-Hydroxytryptophan	HMDB0000472	Yes
ADMA	HMDB0001539	Yes
Alanine	HMDB0000161	Yes
Aminoadipate	HMDB0000510	Yes
Arginine	HMDB0000517	Yes
Asparagine	HMDB0000168	Yes
Aspartate	HMDB0000191	Yes
Betaine	HMDB0000043	Yes
Citrulline	HMDB0000904	Yes
Creatine	HMDB0000064	Yes
Creatinine	HMDB0000562	Yes
Cystathionine	HMDB0000099	Yes
Dimethylglycine	HMDB0000092	Yes
GABA	HMDB0000112	Yes
G-Glutamylcysteine	HMDB0001049	Yes
Glutamate	HMDB0000148	Yes
Glutamine	HMDB0000641	Yes
Glutathione	HMDB0000125	Yes
Glycine	HMDB0000123	Yes
Guanidoacetate	HMDB0000128	Yes
Histidine	HMDB0000177	Yes
Homocysteine	HMDB0000742	Yes
Homoserine	HMDB0000719	-
Hydroxykynurenine	HMDB0000732	Yes
Isoleucine	HMDB0000172	Yes
Kynurenine	HMDB0000684	Yes
Leucine	HMDB0000687	Yes
Lysine	HMDB0000182	Yes
Methionine	HMDB0000696	Yes
Ornithine	HMDB0000214	Yes
Phenylalanine	HMDB0000159	Yes
Proline	HMDB0000162	Yes
SDMA	HMDB0003334	Yes
Serine	HMDB0000187	Yes
Threonine	HMDB0000167	Yes
Tryptophan	HMDB0000929	Yes
Tyrosine	HMDB0000158	Yes
Valine	HMDB0000883	Yes
Benzoic acids and derivatives		
3-Hydroxanthranilate	HMDB0001476	-
Hippurate	HMDB0000714	Yes
Beta amino acids and derivatives		
Carnosine	HMDB0000033	Yes
Pantothenate	HMDB0000210	Yes
Bile acids, alcohols, and derivatives		
Chenodeoxycholate	HMDB0000518	Yes
Cholate	HMDB0000619	Yes
Glycocholate	HMDB0000138	Yes
Taurochenodesoxycholate	HMDB0000951	Yes
Carbohydrates and carbohydrate conjugates		
d-Ribose-5-P	HMDB0001548	Yes
Glyceraldehyde	HMDB0001051	Yes
Sucrose	HMDB0000258	–
Dialkylamines		
Spermidine	HMDB0001257	Yes
Dicarboxylic acids and derivatives		
Succinate	HMDB0000254	Yes
Fatty acyls		
Acetylcarnitine	HMDB0000201	Yes
Decanoylcarnitine	HMDB0000651	Yes
Hexanoylcarnitine	HMDB0000705	Yes
Isobutyrylcarnitine	HMDB0000736	Yes
Isovalerylcarnitine	HMDB0000688	Yes
Octanoylcarnitine	HMDB0000791	Yes
Propionylcarnitine	HMDB0000824	Yes
Glucuronic acid and derivatives		
Glucuronate	HMDB0000127	Yes
Imidazoles		
1-Methylhistamine	HMDB0000898	Yes
Allantoin	HMDB0000462	Yes
Indoles and derivatives		
5-Hydroxyindoleacetate	HMDB0000763	Yes
Keto acids and derivatives		
Acetoacetate	HMDB0000060	Yes
Organic phosphoric acids and derivatives		
Phosphoethanolamine	HMDB0000224	Yes
Organosulfonic acids		
Taurine	HMDB0000251	Yes
Taurocholate	HMDB0000036	Yes
Oxides		
Trimethylamine N-oxide	HMDB0000925	Yes
Phenols		
Homogentisate	HMDB0000130	Yes
Normetanephrine	HMDB0000819	Yes
Pteridines and derivatives		
Neopterin	HMDB0000845	Yes
Purines and derivatives		
Adenine	HMDB0000034	Yes
Adenosine	HMDB0000050	Yes
AMP	HMDB0000045	Yes
cAMP	HMDB0000058	–
cGMP	HMDB0001314	–
Guanosine	HMDB0000133	Yes
Hypoxanthine	HMDB0000157	Yes
IMP	HMDB0000175	Yes
Inosine	HMDB0000195	Yes
Xanthine	HMDB0000292	Yes
Xanthosine	HMDB0000299	Yes
Pyridines and derivatives		
4-Pyridoxate	HMDB0000017	Yes
Cotinine	HMDB0001046	–
NAD	HMDB0000902	Yes
Niacinamide	HMDB0001406	Yes
Nicotinate	HMDB0001488	Yes
Pyridoxine	HMDB0000239	–
Pyrimidines and derivatives		
Cytidine	HMDB0000089	Yes
Cytosine	HMDB0000630	Yes
Deoxycytidine	HMDB0000014	Yes
Deoxyuridine	HMDB0000012	Yes
Orotate	HMDB0000226	Yes
UDP Glucose	HMDB0000286	–
Uracil	HMDB0000300	Yes
Quaternary ammonium salts		
Carnitine	HMDB0000062	Yes
Choline	HMDB0000097	Yes
Quinolines and derivatives		
Kynurenate	HMDB0000715	Yes
Sugar alcohols		
Myo-inositol	HMDB0000211	Yes
Sorbitol	HMDB0000247	Yes

*HMDB ID*, Human Metabolome Database identifier; *ADMA*, asymmetric dimethylarginine; *GABA*, gamma-aminobutyric acid; *SDMA*, symmetric dimethylarginine; *AMP*, adenosine monophosphate; *cAMP*, cyclic adenosine monophosphate; *cGMP*, cyclic guanosine monophosphate; *IMP*, inosine monophosphate; *NAD*, nicotinamide adenine dinucleotide; *UDP*, uridine diphosphate.

**Table E2:** Donor plasma metabolites predicting biopsy-proven acute rejection

Metabolite	Events	Concordance	Hazard ratio (95% CI); *P* value	AUC∗	AUC†
Glycine	7	0.814 ± 0.082	1.02 (1.01-1.02); *P* < .001	0.85	0.81
Glyceraldehyde	7	0.867 ± 0.047	1.04 (1.02-1.06); *P* < .001	0.88	0.86
Phenylalanine	7	0.839 ± 0.057	1.06 (1.03-1.10); *P* = .001	0.85	0.80
Threonine	7	0.812 ± 0.067	1.02 (1.01-1.03); *P* < .001	0.84	0.80
L-Methionine	7	0.776 ± 0.104	1.05 (1.02-1.08); *P* = .001	0.88	0.75
Proline	7	0.791 ± 0.111	1.02 (1.01-1.03); *P* = .001	0.84	0.77
Serine	7	0.787 ± 0.074	1.04 (1.01-1.06); *P* = .002	0.81	0.77
Histidine	7	0.789 ± 0.084	1.02 (1.01-1.04); *P* = .002	0.81	0.85
Asparagine	7	0.741 ± 0.101	1.04 (1.01-1.06); *P* = .002	0.8	0.77
Lysine	7	0.731 ± 0.099	1.01 (1.00-1.02); *P* = .001	0.79	0.83
Arginine	7	0.770 ± 0.103	1.03 (1.01-1.05); *P* = .001	0.77	0.75
Hydroxyproline	7	0.752 ± 0.101	1.09 (1.02-1.17); *P* = .014	0.76	0.82

Values are presented as concordance ± SE, unless otherwise noted.

∗ Time-dependent receiver operating characteristic (t = 365).

† weibull (t = 365).

**Table E3:** Relationship of donor plasma glycine levels with donor demographics

Donor demographic characteristic	High glycine (n = 30)	Low glycine (n = 53)	*P* value
Donor sex			
Female	37 (11)	15 (8)	**.048**
Male	63 (19)	85 (45)	
Donor age	40 ± 13	43 ± 10	.45
Donor height	175 ± 7.8	178 ± 8.7	.24
Donor weight	75 ± 14	82 ± 15	.068
Donor BMI	24 ± 3.5	26 ± 5.0	.081
Donor pulmonary hypertensive markers	171 ± 30	188 ± 28	.053
Donor smoking, current	40 (12)	43 (23)	.94
Donor aspiration	13 (4)	23 (12)	.47
Donor EBV status	97 (29)	89 (47)	.21
Donor CMV status	83 (25)	83 (44)	1
Donor hemoglobin	117 ± 16	123 ± 23	.095
Donor platelets, thrombocytes	171 ± 81	180 ± 79	.81
Donor CRP	44 ± 64	79 ± 87	.19
Donor troponin T	67 ± 91	32 ± 43	.13
Donor troponin I	322 ± 519	180 ± 487	.29
Donor CK-MB	18 ± 30	15 ± 45	.77
Donor total cholesterol	2.5 ± 0.93	2.9 ± 0.91	.21
Donor HDL cholesterol	0.91 ± 0.45	0.99 ± 0.41	.67
Donor LDL cholesterol	1.1 ± 0.67	1.3 ± 0.70	.28
Donor triglycerides	0.79 ± 0.48	0.92 ± 0.52	.29
Donor cause of death			
Gunshot wound	6.7 (2)	3.8 (2)	.71
Intracerebral hemorrhage	17 (5)	23 (12)	
Ischemic brain injury	6.7 (2)	13 (7)	
Subarachnoid hemorrhage	37 (11)	25 (13)	
Subdural hematoma/trauma	27 (8)	32 (17)	
Other	6.7 (2)	3.8 (2)	
Donor ejection fraction	62 ± 6.1	61 ± 5.6	.83
Donor abnormal cardiac motion	13 (4)	5.7 (3)	.44
Donor abnormal coronary angiography	10 (3)	9.4 (5)	1
Donor inotropic support	87 (26)	66 (35)	.68

Values are presented as % (n) or mean ± SD. Boldface in the *P*-value column indicates statistically significant values (*P* < .05). *BMI*, Body mass index; *EBV*, Epstein–Barr virus; *CMV*, cytomegalovirus; *CRP*, C-reactive protein; *CK-MB*, creatine phosphokinase-MB; *HDL*, high-density lipoprotein; *LDL*, low-density lipoprotein.

**Table E4:** Relationship of donor plasma glycine levels with recipient demographics

Recipient demographic characteristics	High glycine (n = 30)	Low glycine (n = 53)	*P* value
Recipient age (y)	54 ± 9.2	53 ± 11	.85
Recipient sex			
Female	37 (11)	21 (11)	.19
Male	63 (19)	79 (42)	
Recipient height	172 ± 10	174 ± 7.7	.51
Recipient weight	73 ± 15	80 ± 18	.39
Recipient BMI	25 ± 3.7	26 ± 4.7	.085
Recipient ECMO support	3.3 (1)	11 (6)	.4
Recipient EBV-positive	97 (29)	91 (48)	.41
Recipient CMV-positive	73 (22)	75 (40)	.49
Recipient hemoglobin	104 ± 15	105 ± 14	.65
Recipient bilirubin	21 ± 19	17 ± 15	.67
Recipient ALAT	32 ± 25	36 ± 46	.74
Recipient GFR	59 ± 18	57 ± 23	.19
Recipient PRA class I	16 ± 29	4.3 ± 12	.075
Recipient PRA class II	4.7 ± 19	3.6 ± 17	.87
Cold ischemia time (min)	94 ± 55	89 ± 52	.99
Warm ischemia time (min)	79 ± 18	81 ± 26	.76
Total ischemia time (min)	174 ± 52	167 ± 63	.77
Perfusion support duration (min)	70 ± 19	87 ± 32	.096
Total intubation time (h)	299 ± 763	121 ± 171	.76
ICU stay (d)	415 ± 548	308 ± 262	.76
Total hospital stay (d)	45 ± 30	40 ± 28	.76
ATG therapy	27 (8)	51 (27)	.055
Cyclosporine therapy	17 (5)	17 (9)	.58
Tacrolimus therapy	80 (24)	74 (39)	.58
Azathioprine therapy	6.7 (2)	3.8 (2)	.51
Mycophenolate therapy	90 (27)	85 (45)	.58
Steroid therapy	97 (29)	91 (48)	.56

Values are presented as mean (SD) or % (n), with *P* values comparing groups. *BMI*, Body mass index; *ECMO*, extracorporeal membrane oxygenation; *EBV*, Epstein–Barr virus; *CMV*, cytomegalovirus; *ALAT*, alanine aminotransferase; *GFR*, glomerular filtration rate; *PRA*, panel reactive antibody; *ICU*, intensive care unit; *ATG*, antithymocyte globulin.

**Table E5:** Recipient characteristics, sensitization markers, and immunosuppression regimens stratified by rejection severity (≤ G1R vs ≥ G2R)

Variable	≤G1R (n = 76)	≥G2R (n = 7)	*P* value
Recipient age (y)	54 ± 11	54 ± 8.8	.46
Recipient sex			.14
Female	24 (18)	57 (4)	
Male	76 (58)	43 (3)	
Height (cm)	174 ± 8.6	169 ± 9.4	.26
Weight (kg)	77 ± 16	79 ± 22	.49
BMI	25 ± 4.4	27 ± 4.6	.36
ECMO support			.9
Yes	9.2 (7)	0 (0)	
No	91 (69)	100 (7)	
Recipient EBV seropositive	93 (71)	86 (6)	.26
Recipient CMV seropositive	74 (56)	86 (6)	.76
Hemoglobin	104 ± 15	106 ± 8.5	.18
Bilirubin	19 ± 17	16 ± 12	.74
ALAT	35 ± 41	33 ± 11	.11
GFR	57 ± 21	64 ± 22	.55
PRA class I	7 ± 16	26 ± 45	.22
PRA ≥20%, class I	12 (9)	29 (2)	.42
PRA class II	3 ± 15	15 ± 38	.08
Cold ischemia time	94 ± 51	65 ± 66	.24
Warm ischemia time	80 ± 24	85 ± 20	.76
Total ischemia time	171 ± 59	149 ± 60	.26
Perfusion support time	83 ± 29	58 ± 17	.11
Intubation time	164 ± 474	417 ± 604	.24
ICU stay	327 ± 370	557 ± 584	.14
Hospital stay (d)	40 ± 26	62 ± 42	.072
ATG induction	43 (33)	29 (2)	.72
Cyclosporine therapy	16 (12)	29 (2)	.55
Tacrolimus therapy	76 (58)	71 (5)	.55
Azathioprine therapy	3.9 (3)	14 (1)	.37
Mycophenolate therapy	87 (66)	86 (6)	.49
Steroid therapy	92 (70)	100 (7)	.99

Values are presented as mean ± SD or % (n), with *P* values comparing groups. *ECMO*, Extracorporeal membrane oxygenation; *EBV*, Epstein–Barr virus; *CMV*, cytomegalovirus; *ALAT*, alanine aminotransferase; *GFR*, glomerular filtration rate; *PRA*, panel reactive antibody; *ICU*, intensive care unit; *ATG*, antithymocyte globulin; *G1R*, grade 1 rejection; *G2R*, grade 2 rejection.

## Results

Plasma levels of 24 metabolites were significantly altered in heart transplant donors (Figure E2). Glycine correlated with extracellular matrix organization-related metabolites proline and hydroxyproline (Figure E3). Comprehensive comparisons of donor and recipient characteristics were performed between the high- and low-glycine groups (Tables E3 and E4). Among donors, no significant differences were observed in established risk factors, including age, cause of death, vasopressor use, left ventricular function, or cold ischemic time, indicating that elevated glycine level is unlikely to represent a surrogate for conventional donor risk parameters. Donor sex showed a borderline difference between groups (*P* = .048); however, adjustment for sex did not attenuate the association between donor glycine levels and rejection at Grade 2 or greater. Recipient characteristics—including age, sex, body mass index, comorbidities, induction therapy, and maintenance immunosuppression—were comparable between groups, suggesting that the observed association is not driven by recipient-related confounding. In addition, recipient-related factors were evaluated according to rejection outcome. Immunosuppression regimens, sensitization status, and key clinical characteristics did not differ between recipients with and without rejection at Grade 2 greater (Table E5). Together, these findings support the interpretation that donor plasma glycine level reflects a biologically distinct signal independently associated with rejection risk.

### Lack of Significant Correlation Between Metabolite Profile and Primary Graft Dysfunction

Out of the 83 recipients, 25 individuals exhibited varying degrees of left ventricular primary graft dysfunction (PGD), and only 8 of them had severe left ventricular PGD cases. When we conducted a comparison of metabolic levels among different grades of PGD, our analysis revealed no statistically significant changes in metabolite profiles between those without PGD and those with PGD, as well as between those with nonsevere PGD and those with severe PGD.

Among the metabolites analyzed, choline and adenosine monophosphate (AMP) showed nominal differences between PGD and No PGD groups; however, after FDR correction, these differences were no longer statistically significant. In our dataset, donor glycine levels did not correlate with PGD (*P* = .85). Logistic regression similarly showed no association between donor glycine concentration and the occurrence of PGD (*P* = .85), and this remained unchanged after adjustment for donor age and cold ischemic time (Figure E4). Donor plasma metabolic profile was associated with biopsy-proven acute rejection of grade ≥G2R within 1 year of transplantation.

In this study, 8.43% of transplant recipients experienced biopsy-proven acute rejection of grade ≥G2R within 1 year of transplantation. Cox regression analysis connected 12 metabolites in donor plasma with this outcome.

## Appendix E1 Methods

### Study Design

We collected plasma samples from 84 brain-dead heart transplant donors in a prospective randomized clinical trial (Donor Simvastatin Treatment in Organ Transplantation (SIMVA); ClinicalTrials.gov ID: NCT01160978) at the Helsinki University Hospital between 2010 and 2016.E1, E2 The Finnish Red Cross Blood Service supplied plasma samples from 20 healthy volunteers who were age- and sex-matched to the study cohort to serve as a control group. In the original trial, donors were randomly assigned at a 1:1 ratio to receive either 80 mg simvastatin or no simvastatin within the first 2 hours after declaration of brain death. Donor plasma samples for metabolomics and proteomics analyses were obtained immediately before organ procurement, with an average of 15 hours (range, 7-36 hours) having passed between simvastatin treatment and donor plasma sampling. Heart transplant left ventricle myocardial biopsies were taken immediately before reperfusion of the heart, with a total median ischemia time of 180 minutes (120-213 minutes). The results of the main study showed that donor simvastatin treatment reduced the plasma levels of postoperative biomarkers for myocardial damage and the number of hemodynamically compromised acute rejections within 30 days after heart transplantation.^E1^ There were no statistically significant differences in demographics between the simvastatin-treated and simvastatin-untreated donors in the trial.

### Donor Plasma Metabolic Profile Remained Unaltered By Donor Simvastatin Treatment

Donor simvastatin treatment was given within the first 2 hours after declaration of brain death and plasma samples were taken from donors immediately before organ procurement. For all donors, the duration between brain death and organ procurement was approximately 15 hours. There were no statistically significant differences in donor demographics between the donors treated with simvastatin and those left untreated.

Our results demonstrate that simvastatin treatment has no effect on the targeted plasma metabolic profile of brain-dead donors before heart procurement (Figure E1). Furthermore, simvastatin treatment of donors was not associated with the development of primary graft dysfunction, acute rejection (biopsy-proven grade 2 rejection [≥G2R]), or graft-related survival after heart transplantation. Therefore, simvastatin treatment was not considered a confounding factor in this study, and as a result, treated and untreated donors were processed as a single cohort in this study.

Donor and heart transplant recipient care and follow-up were performed according to the written clinical protocol of the Heart and Lung Transplantation Program at the Helsinki University Hospital.^E1^ Donors were given 1 g methylprednisolone and of meropenem intravenously immediately after declaration of brain death. At the time of procurement, donor hearts were perfused with Plegisol (Hospira Inc) cardioplegic solution 30 mL/kg body weight.

To validate the results, we used plasma samples from an external validation set of 48 heart transplantations performed between 2019 and 2022 at the Helsinki University Hospital (Biomarkers for Diagnosis, Prognosis, and Targeted Therapy After Heart Transplantation [EMBIO]; ClinicalTrials.gov ID: NCT06064123). EMBIO is a prospective observational single center study to investigate different liquid biopsies as surrogates for endomyocardial biopsy to detect clinically significant acute rejection after heart transplantation. We collected plasma samples from 48 brain-dead heart transplant donors immediately before organ procurement and used donor and recipient demographic data. The SIMVA and EMBIO studies were conducted according to the Principles of the Helsinki Declaration and were reviewed and approved by the Institutional Ethical Committee of the Helsinki University Hospital (permissions: 358/13/03/02/2009 and HUS/3654/2017). In addition, the SIMVA study was reviewed and approved by the Finnish Medicines Agency, Helsinki, Finland.

### Heart Transplant Donor Metabolomic Profile Data Acquisition

We collected blood samples in lithium heparin tubes (Becton Dickinson) for the training set (SIMVA) and in EDTA tubes (Becton Dickinson) for the validation set (EMBIO). The training set plasma samples were prepared by centrifugation at 1600 × g for 10 minutes at room temperature, whereas the validation samples were centrifuged at 2500 × g for 10 minutes at room temperature. In both sets, the plasma samples were stored in cryotubes at −80 °C until use. We analyzed targeted 102 metabolites of the plasma samples with an Acquity (Waters Corporation) ultra-high performance liquid chromatography-mass spectrometry system coupled to a Xevo TQ-S triple quadrupole mass spectrometer (Waters Corporation) according to the manufacturer's instructions.^E3^ The targeted 102 metabolites cover major metabolic pathways from 23 different classes (Table E1).

Briefly, we added 10 μL internal standard mixture to 100 μL sample, followed by extraction solvent, and dispensed the collected supernatant in an Ostro 96-well plate (Waters Corporation). We injected 5 μL vacuum-filtered sample extract into ultra-high performance liquid chromatography-mass spectrometry for metabolite separation and quantification. MassLynx 4.1 software (Waters Corporation) was used for data acquisition, data handling, and instrument control, whereas TargetLynx software (Waters Corporation) was applied for data processing and reporting of quantitative results. To prevent batch effect and technical variation in the data, metabolomic data from brain-dead heart transplant donors and healthy controls were acquired, handled, preprocessed, and normalized together.

### Statistical and Bioinformatics Analyses

The metabolomics data analysis was conducted using MetaboAnalyst^9^ software (version 3.0). First, we examined the data to ensure that all metabolite peaks were incorporated into each sample's peak shape and that their concentrations were above the lower limit of quantification value. Next, the data were normalized using the median approach, log2 transformation, and pareto scaling. To explore the maximum separation among the study groups, unsupervised and supervised multivariate regression techniques were used, specifically principal component analysis (PCA), partial least squares discriminant analysis (PLS-DA), and orthogonal partial least squares discriminant analysis. In PCA, principal components (PCs) are composite variables representing patterns of variation in the metabolite data, with PC 1 capturing the largest variability and PC 2 the second largest; component 1 and component 2 correspond to PC 1 and PC 2 and are shown on the *x*- and *y*-axes in PCA plots to illustrate sample clustering. In PLS-DA, the T score represents each sample's position along a latent variable that best separates the groups, with higher or lower values reflecting how strongly a sample expresses the associated metabolic pattern. The metabolites were ranked based on their variable importance of projection scores in the PLS-DA model. Metabolites with variable importance of projection scores >1 were considered the most significant contributors. To investigate differences among the groups, univariate analyses were performed on the normalized data, including fold-change analysis and *t* tests. The metabolites were ranked based on their significance level using a volcano plot with an FC threshold of 1 and a false discovery rate (FDR) -corrected *t* test *P* value threshold of .05.

In this study, MetaboAnalyst software was used to investigate the biological pathways and annotations for significant metabolites. First, human metabolome database identifications for metabolites was queried, followed by overrepresentation analysis using the Kyoto Encyclopedia of Genes and Genomes database to investigate whether metabolites from predefined sets (Kyoto Encyclopedia of Genes and Genomes pathway database last accessed September 14, 2023) are more abundant than one might expect within a subset of data. To rule out the possibility of selection bias in targeted metabolites, we performed an overrepresentation analysis on 93 identified metabolites as a reference set. Also, disease ontology enrichment analysis was performed to find significant diseases associated with the differentially expressed metabolites utilizing the Disease Ontology Semantic and Enrichment database.

For the prediction analysis, data on brain-dead donor metabolites were analyzed to figure out their association with the risk of developing biopsy-proven acute rejection (grade 2 rejection [≥G2R]) within 1 year after heart transplantation. We used Cox regression (Cox proportional hazard model) analysis, a commonly used statistical method for analyzing time-to-event data (acute rejection with ≥G2R). We included metabolite levels, donor age, and recipient age as prediction factors in our model. We then assessed the quality of our prediction model using three metrics: concordance, area under the curve, and FDR-corrected *P* value. We set our cutoff values to be ≥ 0.8 for concordance and area under the curve and <0.05 for FDR-corrected *P* value to determine the quality of our model. All statistical analyses were performed using R software (R Foundation for Statistical Computing) using packages such as *survival*, *tdROC*, *survminer*, *survivalROC*, *ggplot*, and *finalfit*.

We also used maximally selected rank statistics algorithms, which is a nonparametric method for finding the best cut points for continuous variables. This allowed us to divide the donors into high- and low-level expression subgroups for investigated metabolites. The best cut point is defined as the point at which the 2 subgroups have the largest difference in their Kaplan-Meier curves. By finding these cut points, we figured out the threshold values of those metabolites that were most predictive of acute rejection. We also plotted the receiver operating characteristic curve and cumulative hazard to visualize the performance of the prediction model. High glycine was defined as concentrations above the cohort median (218 μM), and the optimal *maxstat*-derived threshold closely aligned with this median-based categorization.

To investigate the relationship between glycine levels and the various other metabolites in plasma, we performed correlation analysis using the Pearson correlation coefficient. In addition, we performed correlation analysis to investigate the relationship between glycine levels and donor and recipient demographic factors, which included both categorical and continuous variables. We used the *corrplot* package in R software to visualize the correlation matrix. For continuous variables, we calculated the Pearson correlation coefficient, whereas for categorical variables, we used the point-biserial correlation coefficient.
